# Superconducting H_5_S_2_ phase in sulfur-hydrogen system under high-pressure

**DOI:** 10.1038/srep23160

**Published:** 2016-03-17

**Authors:** Takahiro Ishikawa, Akitaka Nakanishi, Katsuya Shimizu, Hiroshi Katayama-Yoshida, Tatsuki Oda, Naoshi Suzuki

**Affiliations:** 1 Center for Science and Technology under Extreme Conditions, Graduate School of Engineering Science, Osaka University, 1–3 Machikaneyama, Toyonaka, Osaka 560-8531, Japan; 2 Graduate School of Engineering Science, Osaka University, 1-3 Machikaneyama, Toyonaka, Osaka 560-8531, Japan; 3 Institute of Science and Engineering, Kanazawa University, Kakuma, Kanazawa, Ishikawa 920-1192, Japan; 4 Department of Pure and Applied Physics, Kansai University, 3-3-35 Yamate, Suita, Osaka 564-8680, Japan

## Abstract

Recently, hydrogen sulfide was experimentally found to show the high superconducting critical temperature (*T*_c_) under high-pressure. The superconducting *T*_c_ shows 30–70 K in pressure range of 100–170 GPa (low-*T*_c_ phase) and increases to 203 K, which sets a record for the highest *T*_c_ in all materials, for the samples annealed by heating it to room temperature at pressures above 150 GPa (high-*T*_c_ phase). Here we present a solid H_5_S_2_ phase predicted as the low-*T*_c_ phase by the application of the genetic algorithm technique for crystal structure searching and first-principles calculations to sulfur-hydrogen system under high-pressure. The H_5_S_2_ phase is thermodynamically stabilized at 110 GPa, in which asymmetric hydrogen bonds are formed between H_2_S and H_3_S molecules. Calculated *T*_c_ values show 50–70 K in pressure range of 100–150 GPa within the harmonic approximation, which can reproduce the experimentally observed low-*T*_c_ phase. These findings give a new aspect of the excellent superconductivity in compressed sulfur-hydrogen system.

Search for room-temperature superconductors is a challenging study in materials science, and pressurization and hydrogenation have been considered as a way to push up the superconducting critical temperature, *T*_c_, to the higher region^1^. Recently, high *T*_c_ superconductivity was experimentally observed in compressed hydrogen sulfide (H_2_S) and *T*_c_ reaches 203 K at 150 GPa^2^,^3^, which exceeds copper oxide superconductors^4^,^5^ and sets a record for the highest *T*_c_. In the experiments, the superconducting *T*_c_ shows 30–70 K in pressure range of 100–170 GPa for the H_2_S samples loaded at 100–150 K and compressed to 100 GPa (low-*T*_c_ phase) and increases to 203 K for the samples annealed by heating it to room temperature at pressures above 150 GPa (high-*T*_c_ phase). The pressure-temperature path dependence of *T*_c_ is considered to be involved by changes of stoichiometry from H_2_S, but the details have not been identified completely. Therefore, further studies on sulfur-hydrogen system under high-pressure are required for the understanding of the mechanism for its excellent superconductivity.

Stoichiometry, crystal structure, and superconductivity of sulfur-hydrogen system under high-pressure have been investigated by first-principles calculations based on the density functional theory (DFT)^6^,^7^,^8^,^9^,^10^,^11^,^12^,^13^,^14^,^15^,^16^,^17^. Duan *et al.* predicted that H_2_S is stable below 43 GPa and decomposes into H_3_S and S above the pressure^8^. For H_2_S, a monoclinic *P*2/*c* structure transforms into a monoclinic *Pc* structure at 27 GPa. H_3_S shows the sequence of pressure-induced structural phase transitions as follows: triclinic *P*1 → orthorhombic *Cccm* (37–111 GPa) → trigonal *R*3*m* (111–180 GPa) → cubic *Im*-3*m*. Other hydrogen-rich stoichiometries, H_4_S, H_5_S, and H_6_S, were reported to be unstable up to at least 300 GPa^8^. Errea *et al.* predicted the stabilizations of HS_2_ above 200 GPa and HS above 300 GPa by including zero-point energy^9^. HS_2_ crystallizes in a monoclinic *C*2/*c* and it transforms into a monoclinic *C*2/*m* above 250 GPa. HS takes a monoclinic *C*2/*m*. Recently, energetically competitive stoichiometries, H_2_S_3_, H_3_S_2_, and H_4_S_3_ were identified^14^. H_2_S_3_ and H_3_S_2_ are unstable above 25 and 34 GPa, respectively, whereas H_4_S_3_ is thermodynamically stable in pressure range from 25 to 113 GPa, in which an orthorhombic P2_1_2_1_2_1_ structure is formed in pressure range of 25–60 GPa and an orthorhombic *Pnma* in 60–113 GPa.

For the superconductivity, *T*_c_ calculated for *Im*-3*m* H_3_S with the inclusion of anharmonic effects shows 194 K at 200 GPa^9^,^10^, which is in good agreement with recent results of synchrotron x-ray diffraction measurements combined with electrical resistance measurements for the high-*T*_c_ phase^18^. On the other hand, for *R*3*m* H_3_S stabilized below 180 GPa, the calculated *T*_c_ is lower by approximately 50 K than the experimentally observed one^9^,^10^,^14^. For the low-*T*_c_ phase, H_2_S has been considered as a candidate, and *T*_c_ is predicted to be 30–60 K for pressure range of 130–160 GPa in a triclinic *P*-1 structure and 80–40 K for 160–250 GPa in an orthorhombic *Cmca*^6^,^11^. However, the calculated *T*_c_ dose not completely reproduce the experimental data in the pressure region except for 150–160 GPa. For other stoichiometries, the calculated *T*_c_ values of *C*2/*c* HS_2_, *C*2/*m* HS_2_, *C*2/*m* HS, and *Pnma* H_4_S_3_ are 23.4 K for the effective screened Coulomb repulsion constant μ^*^ of 0.16 at 200 GPa^9^, 14.9 K at 250 GPa^9^, 23.4 K at 300 GPa^9^, and 0.75 K at 300 GPa^14^, respectively, which are all far from the experimentally observed values.

## Results

**Figure 1:**
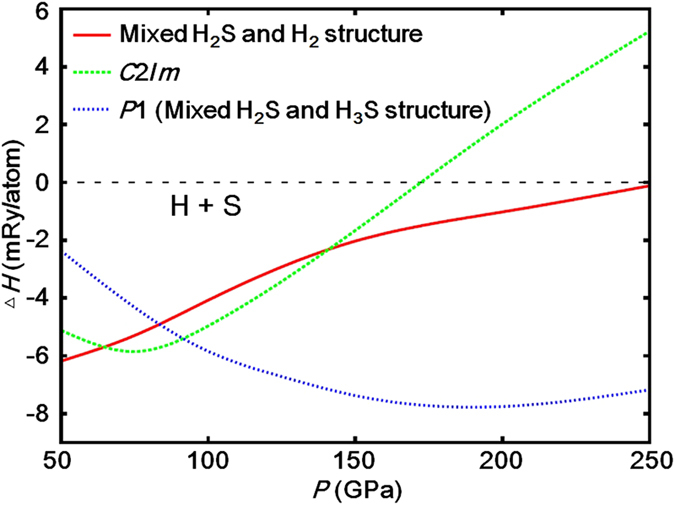
Formation enthalpy per atom, *H*, of crystal structures obtained by the application of the GA technique to the H_5_S_2_ stoichiometry. H_5_S_2_ takes a disorderly mixed structure of H_2_S and H_2_ molecules at 50 GPa, and it transforms into a monoclinic *C*2/*m* at 64 GPa and then into a triclinic *P*1 with mixed H_2_S and H_3_S structure at 92 GPa.

**Figure 2:**
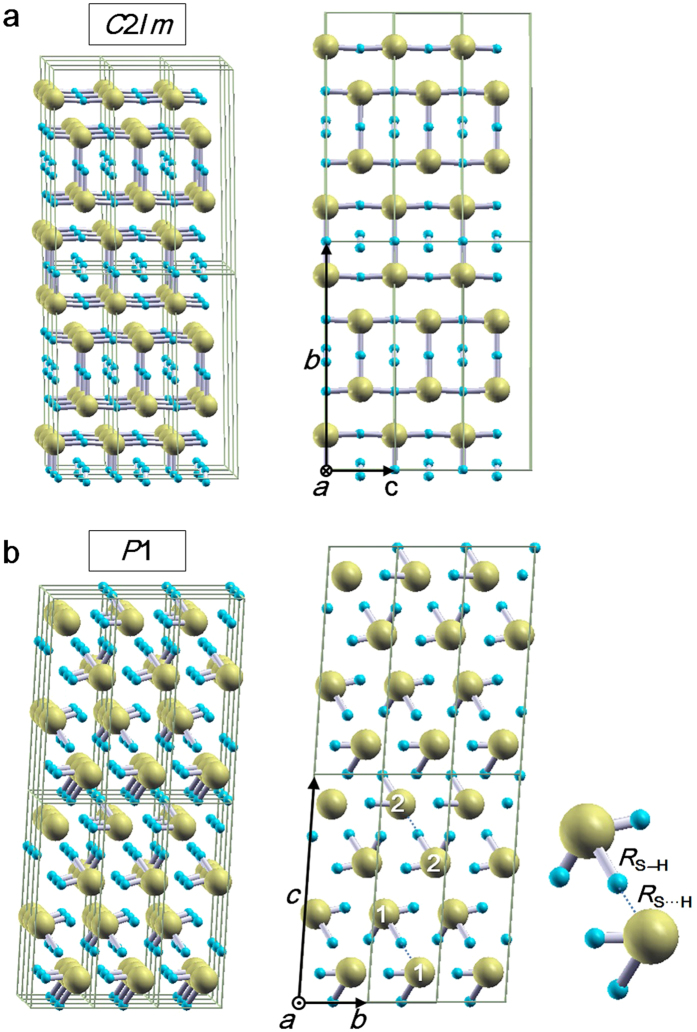
Crystal structures obtained by the GA technique. (**a**) A monoclinic *C*2/*m*. The lattice parameters at 75 GPa are as follows: *a* = 2.1813Å, *b* = 10.5741Å, *c* = 3.0907Å, and *γ* = 77.563°. The S atoms occupy a 4i site with (0.1420, 0.8507, 0), and the H atoms a 2a site and two 4i sites with (0.4323, 0.4710, 0) and (0.1476, 0.3429, 0). (**b**) A triclinic *P*1 with a mixed structure of H_2_S and H_3_S molecules. The lattice parameters at 112 GPa are *a* = 2.7127Å, *b* = 2.7119Å, *c* = 8.6105Å, *α* = 84.706°, *β* = 84.447°, and *γ* = 71.868°. All the atoms occupy 1a sites: (0.1869, 0.1539, 0.3865), (0.2812, 0.2819, 0.8685), (0.7196, 0.7232, 0.1301), and (0.8252, 0.8495, 0.6114) for S, and (0.6031, 0.5675, 0.7345), (0.7238, 0.8394, 0.8735), (0.1554, 0.2735, 0.1260), (0.0269, 0.9946, 0.9939), (0.7166, 0.5739, 0.4144), (0.2905, 0.4248, 0.5862), (0.0626, 0.0306, 0.7276), (0.9602, 0.9548, 0.2744), (0.4342, 0.4268, 0.2592), and (0.5163, 0.4870, 0.0020) for H. Asymmetric hydrogen bonds are formed between the H_2_S and H_3_S molecules: (1) *R*_S–H_ = 1.4886Å and *R*_S_._H_ = 1.5929Å, and (2) *R*_S–H_ = 1.4441Å and *R*_S_._H_ = 1.6596Å, where the numbers in parenthesis correspond to those represented on the S atom and *R* shows the distance between S and H.

In the present study, we focus on another stoichiometric compound, H_5_S_2_, which has hydrogen content between H_2_S and H_3_S. We first explored stable structures of H_5_S_2_ by the genetic algorithm (GA) technique and first-principles calculations based on DFT, and then compared formation enthalpy per atom among the obtained structures in pressure range from 50 to 250 GPa (Fig. 1). The formation enthalpy was calculated as follows: △*H* = *H*_H5S2_−5/7*H*_H_−2/7*H*_S_, where *H*_H5S2_, *H*_H_, and *H*_S_ are the enthalpies per atom of H_5_S_2_, pure hydrogen (H), and pure sulfur (S), respectively. At 50 GPa, H_5_S_2_ takes a disorderly mixed structure of H_2_S and H_2_ molecules. At 64 GPa, a monoclinic *C*2/*m* structure is stabilized. In this structure, four H atoms form two H_2_ molecules, and the other H atoms make covalent bonds with the S atoms (Fig. 2a). The *C*2/*m* structure transforms into a triclinic *P*1 structure at 92 GPa. In this phase, H_5_S_2_ takes a mixed structure of H_2_S and H_3_S molecules, between which asymmetric hydrogen bonds are formed (Fig. 2b). This structure is also interpreted as a member of the Magnéli-like crystals reported very recently^19^.

**Figure 3:**
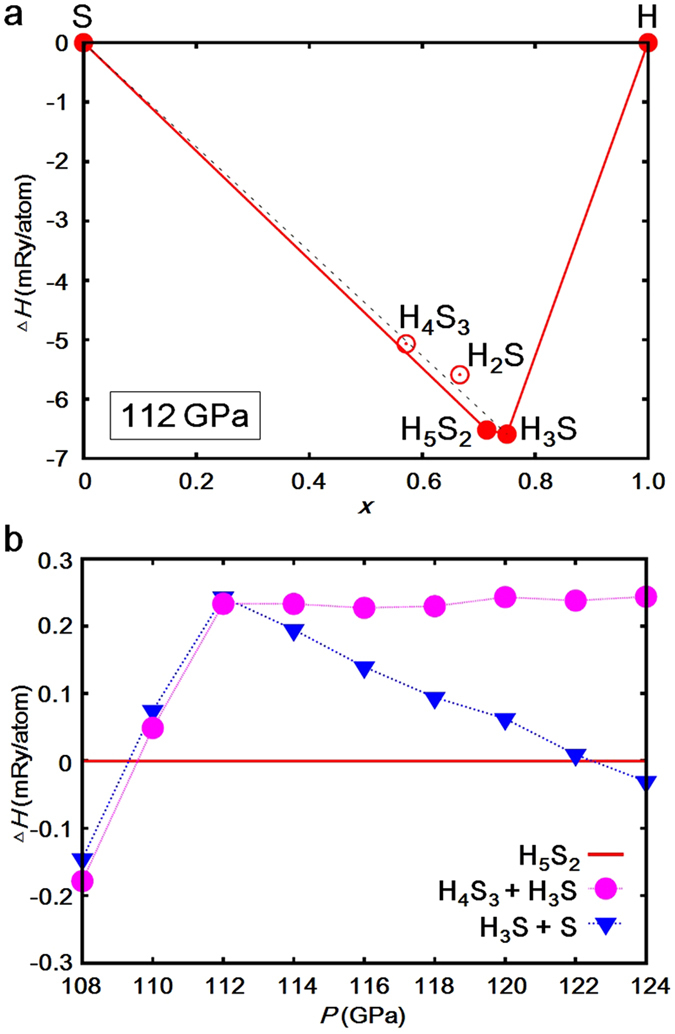
Formation enthalpy per atom as a function of *x* for H_*x*_S_1−*x*_ compounds. a, The formation enthalpies of H_4_S_3_, H_2_S, H_5_S_2_, and H_3_S compounds at pressure of 112 GPa. The H_5_S_2_ and H_3_S compounds are on a convex hull shown by solid line, which indicates that the compounds are thermodynamically stable at this pressure. The other compounds are above the convex hull (open circle), which are predicted to be decomposed. b, Pressure dependence of the enthalpies for H_5_S_2_, H_4_S_3_ + H_3_S, and H_3_S + S. H_5_S_2_ is composed from H_4_S_3_ and H_3_S at 110 GPa (H_4_S_3_ + 7H_3_S → 5H_5_S_2_) and is decomposed into H_3_S and S at 123 GPa (3H_5_S_2_ → 5H_3_S + S).

Next, we investigated thermodynamic stability of H_5_S_2_ from the convex hull diagram with respect to the H-S stoichiometric compounds, H_*x*_S_1−*x*_. Figure 3a shows the static formation enthalpy per atom of the compounds as a function of *x*, which is defined as Δ*H* (*x*) = *H*(*x*)−*xH*_H_−(1−*x*)*H*_S_, at 112 GPa. The structures of the compounds are as follows: β-Po for S 20, *Pnma* for H_4_S_3_^14^, *P*-1 for H_2_S^6^, *P*1 for H_5_S_2_, *R*3*m* for H_3_S^7^, and *B*2/*n* for H^21^. The results show that H_4_S_3_ and H_5_S_2_ are below the line connecting between H_3_S and S (dotted line) but H_4_S_3_ is above the line connecting between H_5_S_2_ and S. As the results, H_5_S_2_ and H_3_S are the compounds on the convex hull (solid line) and are thermodynamically stable at 112 GPa, whereas H_4_S_3_ and H_2_S are predicted to be decomposed at this pressure. Figure 3b shows the formation enthalpies of H_4_S_3_ + H_3_S and H_3_S + S relative to that of H_5_S_2_ as a function of pressure. H_5_S_2_ is composed from H_4_S_3_ and H_3_S at 110 GPa (H_4_S_3_ + 7H_3_S → 5H_5_S_2_), and is decomposed into H_3_S and S at 123 GPa (3H_5_S_2_ → 5H_3_S + S).

**Figure 4:**
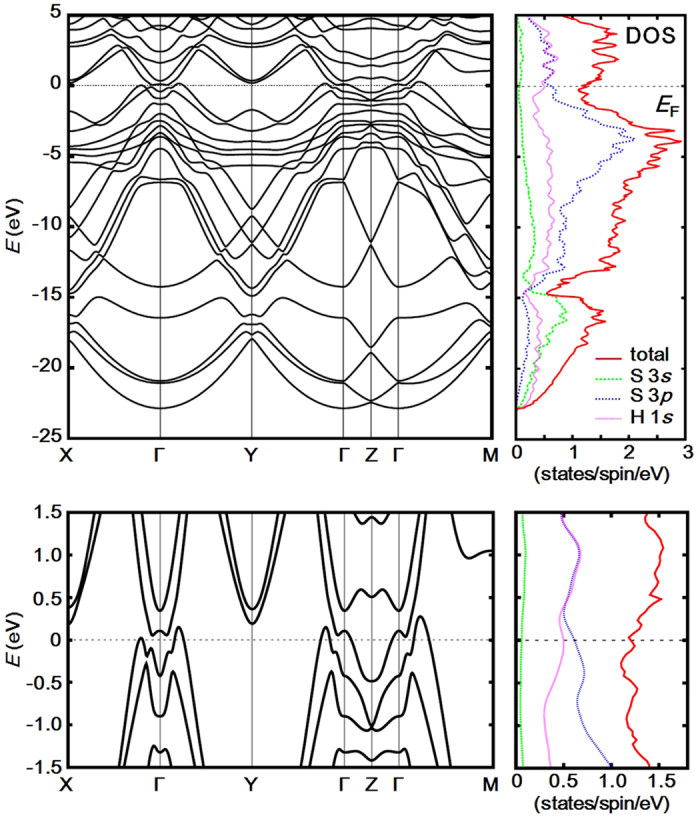
Electronic band structure and density of states (DOS) for *P*1 H_5_S_2_ at 112 GPa. The lower panel shows a close-up view around the Fermi level (*E*_F_). The calculated band structure forms flat bands near *E*_F_ at around the Г symmetry point and H_5_S_2_ is a good metal. The partial DOS shows that S 3*p* and H 1*s* are strongly hybridized at *E*_F_.

Figure 4 shows electronic band structure and density of states (DOS) for *P*1 H_5_S_2_ at 112 GPa. Antibonding bands formed by hybridization of S 3*p* and H 1*s* states, pushed up in the energy owing to the high pressure, appear as flat band dispersions at the Fermi level (*E*_F_) at around the Г symmetry point by overlapping conduction bands. As the results, DOS at *E*_F_, *i.e. N*(*E*_F_), is increased and H_5_S_2_ is a good metal. The hybridization is remarkable at *E*_F_, similar to the cases of H_2_S and H_3_S predicted earlier^6^,^7^,^12^, whereas for a ratio of the H 1*s* state to *N*(*E*_F_) there is a clear distinction among the compounds: *N*_H_(*E*_F_) /*N*(*E*_F_) ≈ 0.50 for H_3_S at 130 GPa^7^, *N*_H_(*E*_F_)/*N*(*E*_F_) ≈ 0.45 for H_5_S_2_ at 112 GPa, and *N*_H_(*E*_F_) /*N*(*E*_F_) ≈ 0.25 for H_2_S at 130 GPa^6^. Therefore, H_5_S_2_ and H_3_S show a larger contribution of H to the electrons at *E*_F_ than H_2_S.

We investigated the superconductivity of metallic *P*1 H_5_S_2_ within the harmonic approximation. Table 1 lists electron-phonon coupling constant *λ*, logarithmic-averaged phonon frequency *ω*_log_, and *T*_c_ calculated for *P*1 H_5_S_2_. The calculated *T*_c_ values show 70.1–79.1 K for μ^*^ = 0.13 and 58.3–66.5 K for μ^*^ = 0.17 in pressure region of 112–130 GPa, which is close to the data for the experimentally observed low-*T*_c_ phase. We also list the similar data for *P*-1 H_2_S^6^,^11^ at 130 GPa considered as a candidate of the low-*T*_c_ phase. The *ω*_log_ value of *P*1 H_5_S_2_ is almost identical to that of *P*-1 H_2_S, whereas the *λ* value *P*1 H_5_S_2_ is almost 1.5 times as large as that of *P*-1 H_2_S. The larger *λ* value is considered to be involved by the larger contribution of H to the electrons at *E*_F_ as mentioned above. As the results, *T*_c_ of *P*1 H_5_S_2_ is higher by approximately 40 K than that of *P*-1 H_2_S.

**Figure 5:**
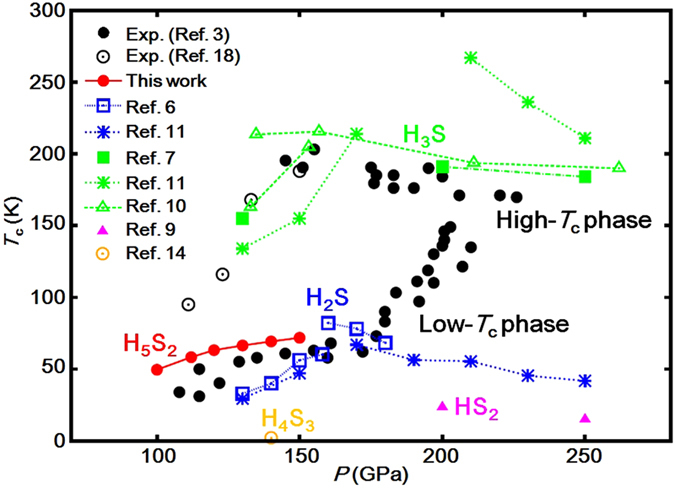
Superconducting critical temperature *T*_c_ of the H_5_S_2_, H_2_S, H_3_S, H_4_S_3_, and HS_2_ compounds. For H_5_S_2_, *T*_c_ is obtained by the Allen-Dynes formula with the effective screened Coulomb repulsion constant μ^*^ of 0.17. Calculated *T*_c_ data of the other compounds and the experimental data are taken from refs 3, 6, 7, 9, 10, 11, 14 and 18, respectively. The H_5_S_2_ phase reproduces the experimental data below 170 GPa in the low-*T*_c_ phase.

**Figure 6:**
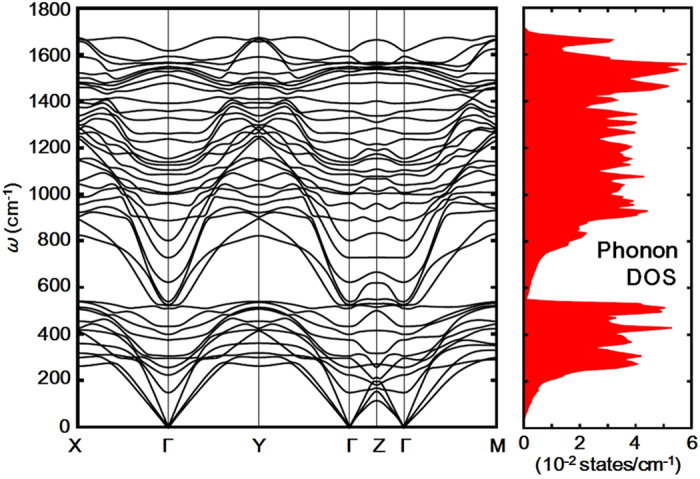
Phonon dispersion curves and density of states for *P*1 H_5_S_2_ at 150 GPa. *P*1 H_5_S_2_ is thermodynamically unstable above 122 GPa but is mechanically stable to at least 150 GPa.

We plotted the *T*_c_ values obtained at μ^*^ = 0.17 for *P*1 H_5_S_2_ with the earlier-predicted *T*_c_ data for the H_2_S^6^,^11^, H_3_S^7^,^10^,^11^, H_4_S_3_^14^, and HS_2_^9^ compounds and compared it with the experimental data^3^ (Fig. 5). Phonon calculations indicate that *P*1 H_5_S_2_ is mechanically stable to at least 150 GPa (Fig. 6) and is expected to sustain as a metastable phase above 122 GPa owing to high kinetic barrier, rigid grain boundary, *etc.* The *T*_c_ value shows 49.5 K at 100 GPa, slightly increases with pressurization, and reaches 71.8 K at 150 GPa, which are in good agreement with the experimental data below 170 GPa in the low-*T*_c_ phase. These results suggest that *P*1 H_5_S_2_ is a better candidate for the low-*T*_c_ phase than H_2_S, H_4_S_3_, and HS_2_.

## Discussion

We searched for stable crystal structures of the H_5_S_2_ stoichiometry, which has hydrogen content between H_2_S and H_3_S, by the genetic algorithm technique, and found three structures in pressure range from 50 to 250 GPa. H_5_S_2_ takes the disorderly mixed structure of H_2_S and H_2_ molecules below 64 GPa, and it transforms into the monoclinic *C*2/*m* structure. By further compression, the triclinic *P*1 structure with asymmetric hydrogen bonds formed between H_2_S and H_3_S emerges above 92 GPa. The convex hull diagram with respect to the H-S compounds shows that *P*1 H_5_S_2_ is thermodynamically stable in the pressure region from 110–123 GPa. By further compression, *P*1 H_5_S_2_ is predicted to decompose into *R*3*m* H_3_S and S: 3 H_5_S_2_ → 5H_3_S + S.

We calculated the superconducting *T*_c_ for *P*1 H_5_S_2_ and obtained the values of 49.5–71.8 K in pressure region of 100–150 GPa within the harmonic approximation, which shows better superconductivity than *P*-1 H_2_S owing to the large contribution of the H 1*s* electrons to *N*(*E*_F_). Errea *et al.* reported the importance of the anharmonicity in sulfur hydrides under high-pressure^9^,^10^. The anharmonicity hardens H-S stretching modes and softens H-S bending modes, which causes a suppression of *λ*. As the results, *T*_c_ is decreased by approximately 20% (see Table II in ref. 9). In addition, Akashi *et al.* nonempirically determined μ^*^ as 0.155 for H_2_S and 0.168 for H_3_S at 130 GPa^11^. Considering these earlier works, we expect that *T*_c_ can be corrected to 46.6 K at 112 GPa and 53.2 K at 130 GPa in *P*1 H_5_S_2_, which are in good agreement with the experimental data for the low-*T*_c_ phase (see Table 1). Our predicted *P*1 H_5_S_2_ is mechanically stable to at least 150 GPa, whereas imaginary phonon frequency appears above the pressure. However, the stable region of the *P*1 H_5_S_2_ phase is expected to be shifted in higher pressure with the inclusion of the anharmonic effects because the phonon frequency is reported to be pushed up in H_3_S owing to the anharmonic effects^9^,^10^. Therefore, the sharp increase of *T*_c_ experimentally observed above 170 GPa in the low-*T*_c_ phase^2^,^3^ is probably involved by the structural phase transition from *P*1 or the decomposition of H_5_S_2_ into H_3_S via the intermediate Magnéli phases^19^, *i.e.* the transition from the low-*T*_c_ phase into the high-*T*_c_ phase.

**Figure 7:**
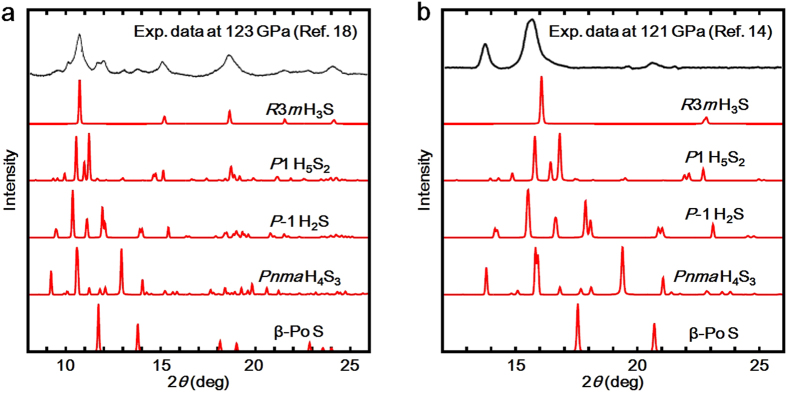
Comparison of x-ray diffraction (XRD) patterns. (**a**) The XRD patterns at 123 GPa are simulated for *R*3*m* H_3_S, *P*1 H_5_S_2_, *P*-1 H_2_S, *Pnma* H_4_S_3_, and β-Po S with the wavelength *λ* of 0.41397Å. The experimental data is taken from Fig. 2a of ref. 18, which is obtained by decompression at room temperature after the high-*T*_c_ phase is observed at 150 GPa. (**b**) The XRD patterns at 121 GPa are simulated with *λ* of 0.6199Å. The experimental data is taken from Fig. 3(a) of ref. 14, which is obtained by compression from 10 GPa at room temperature.

Recently, the x-ray diffraction (XRD) measurements were carried out for H-S system under high-pressure, and *R*3*m* H_3_S and β-Po S was experimentally confirmed in the high-*T*_c_ phase showing *T*_c_ = 203 K^18^. In the experimental XRD patterns, however, there are some peaks which cannot be identified only by *R*3*m* H_3_S and β-Po S^18^. We checked whether the unidentified peaks are obtained by our predicted H_5_S_2_ compound. Figure 7a shows XRD patterns of *R*3*m* H_3_S, *P*1 H_5_S_2_, *P*-1 H_2_S, *Pnma* H_4_S_3_, and β-Po S at 123 GPa, simulated by RIETAN-2000^22^. The experimental XRD pattern was taken from Fig. 2a of ref. 18, which is obtained by decompression at room temperature after the high-*T*c phase is observed at 150 GPa. The wavelength *λ* of 0.41397 Å was employed for the simulated XRD patterns, which is the same value as the experimental data. The XRD pattern of *P*1 H_5_S_2_ shows small diffraction peaks at around 2*θ* = 10° and at 2*θ* = 13°, which seem to correspond to the unidentified peaks observed in the experimental XRD pattern. However, we cannot clearly conclude that the experimental sample contains *P*1 H_5_S_2_ owing to the overlapping of many peaks. We also compared the simulated XRD patterns with another experimental data at 121 GPa, taken from Fig. 3(a) of ref. 14 (Fig. 7b). The experimental data is obtained by compression from 10 GPa at room temperature, which is a different experimental protocol from ref. 18. The wavelength *λ* was set at 0.6199 Å. *P*1 H_5_S_2_ seems to be included in the experimental XRD data with *Pnma* H_4_S_3_, while it is hard to explicitly claim the existence of H_5_S_2_ owing to weak intensity for small peaks in the experimental data. As noted in refs 3 and 14, dissociation products of H_2_S depend on pressure-temperature paths. Therefore, the H_5_S_2_ compound could be observed by further experimental search for optimum pressure-temperature paths to the low-*T*_c_ phase.

## Methods

We searched for crystal structures by our genetic algorithm (GA) code, which has been developed along the line proposed by Glass *et al.*^23^ and been combined with the Quantum ESPRESSO (QE) code^24^. In our GA search, we carried 20 structures in each generation, where 8 structures are created by “crossover”, 6 “mutation”, and 6 “permutation”. We used the simulation cells including 1 to 4 formula units for H_5_S_2_. Pressures are set at 100, 150, and 200 GPa. The generalized gradient approximation by Perdew, Burke and Ernzerhof^25^ was used for the exchange-correlation functional, and the Vanderbilt ultrasoft pseudopotential^26^ was employed. The energy cut-off of the plane wave basis was set at 80 Ry. Marzari-Vanderbilt smearing^27^ with the width of 0.01 Ry was used for the calculations and the *k*-space integration over the Brillouin zone (BZ) was performed on an 8 × 8 × 8 grid. We increased the number of k-points to a 16 × 16 × 16 grid for the most stable structures obtained through the GA search, which is enough to achieve a convergence within 0.1 mRy/atom in the enthalpy at each pressure.

The superconducting *T*_c_ was calculated by the use of the Allen-Dynes formula^28^.


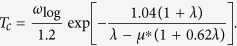


The parameters of electron-phonon coupling constant *λ* and logarithmic-averaged phonon frequency *ω*_log_ represent a set of characters for the phonon-mediated superconductivity. The softening on the phonon mode is induced by a strong electron-phonon interaction, *i.e.* large *λ*, resulting in a decrease of *ω*_log_. Therefore, *T*_c_ is determined by a balance between *λ* and *ω*_log_. Using the QE code, we calculated these parameters with a 4 × 4 × 4 *q*-point grid. The *k*-space integration over BZ was performed on a 16 × 16 × 8 grid, and the electron-phonon matrix element at each *q*-point was calculated by a 32 × 32 × 16 grid. The effective screened Coulomb repulsion constant μ^*^ was assumed to be 0.13–0.17, which is the values nonempirically determined by DFT for superconductors in sulfur-hydrogen system (see Table VI in ref. 11).

## Additional Information

**How to cite this article**: Ishikawa, T. *et al.* Superconducting H_5_S_2_ phase in sulfur-hydrogen system under high-pressure. *Sci. Rep.* **6**, 23160; doi: 10.1038/srep23160 (2016).

## Figures and Tables

**Table 1:** Superconductivity of *P*1 H_5_S_2_.

	*P* (GPa)	*λ*	*ω*_log_ (K)	μ^*^	*T*_c_ (K)	
H_5_S_2_	112	1.1856	898	0.13	70.1	
				0.17	58.3	(46.6)
	120	1.2390	907	0.13	75.2	
				0.17	63.2	(50.6)
	130	1.2417	951	0.13	79.1	
				0.17	66.5	(53.2)
H_2_S	130	0.77	950	0.13	33	
		0.801	913	0.155	29.4	(23.5)
Exp.	115				30, 50	
	122				40	
	130				55	

Superconducting parameters calculated for *P*1 H_5_S_2_ within the harmonic approximation: electron-phonon coupling constant *λ*, logarithmic-averaged phonon frequency *ω*_log_, and superconducting critical temperature *T*_c_. Effective screened Coulomb repulsion constant μ^*^ is assumed to be 0.13 − 0.17. The calculated data for H_2_S and experimental data are taken from refs 3, 6 and 11, respectively. The values in parentheses show the *T*_c_ values decreased by 20%, which are expected in the calculations with the inclusion of the anharmonic effect.
